# Development of an Industrial Process for the Recovery of Palladium from Electroplating Waste Streams Using Polyelectrolyte-Functionalized Adsorbent Textiles

**DOI:** 10.3390/molecules31214010

**Published:** 2026-11-06

**Authors:** Tim Werbeck, David Zapf, Klaus Opwis

**Affiliations:** 1 Hansgrohe SE, Klaus-Grohe-Straße 5-9, 77761 Schiltach, Germany; david.zapf@hansgrohe.com; 2 Deutsches Textilforschungszentrum Nord-West gGmbH (DTNW), Adlerstr. 1, 47798 Krefeld, Germany

**Keywords:** electroplating, palladium recovery, adsorbent textiles, urban mining

## Abstract

In electroplating processes, palladium makes plastic surfaces conductive for subsequent metal plating. During the production process, small amounts of this precious metal end up in the rinse water. Due to the very low palladium concentrations in the presence of significant amounts of interfering ions, recovering excess palladium from these waste streams using conventional methods is labour-intensive and expensive. An alternative is the use of polyelectrolyte-functionalized adsorbent textiles, which are capable of concentrating palladium from aqueous solutions. Through consistent further development of the associated process technology, it is now possible to selectively adsorb palladium from rinse water with very low concentrations—less than 1 mg/L—even in the presence of a high tin load of more than 200 mg/L, and to recover it. As a result, at our production facility in Germany, approximately 5 kg of palladium—currently with a material value of about $200,000—can be recovered annually from process water and returned to the raw material cycle. At the same time, compared to primary palladium production, this achieves a reduction potential of about 140 metric tons of CO_2_ equivalents per year. Our R&D work highlights the significant economic and environmental potential of closed-loop raw material cycles in industrial production processes. Furthermore, this approach offers prospects for additional applications in surface engineering and the recovery of critical raw materials.

## 1. Introduction

The recovery of economically important raw materials, alongside the import of primary raw materials, plays a key role in meeting companies’ demand for raw materials. Particularly in the case of raw materials that are in limited supply and command high prices—such as the strategically and technologically important metals indium and gallium, as well as platinum group metals—effective recovery is not only economically attractive but also conserves resources. According to the European Commission, EU countries are 96% dependent on imports for platinum group elements [1].

As technological metals occur only in limited quantities and their extraction is usually very costly, returning them to the raw materials cycle is a key aspect of meeting global demand. In addition to the recycling of scrap metal, the treatment of process water is also an important source for the recovery of secondary raw materials. However, the composition of process water can vary considerably, and it usually contains only small quantities of valuable metals, which often makes effective treatment and the selection of the appropriate process difficult.

In the field of surface treatment of plastic substrates, surface activation by palladium is essential for the electroplating of non-conductive plastics [2]. The goal of activation is to create electrical conductivity on the substrate surface. The activation solution used consists of colloidal palladium/tin complexes. In addition to metallic components, the activator solution also contains PdCl_2_, SnCl_2_, and HCl. The hydrochloric acid is intended to prevent agglomeration of the particles by forming a chloride-ion-based electrochemical double layer. The amount of palladium adsorbed onto the substrate surface is a key parameter for the successful electroplating of plastic parts. If insufficient palladium is bound to the substrate surface, defects may occur in the coating. The direct metallisation process requires palladium concentrations of 40–60 mg/L in the activation bath [3].

To prevent any carry-over from the upstream process baths into the downstream baths, the activation bath is followed by a rinse stage. There, the racks are washed using immersion and spray methods. A freshly prepared rinse bath consists of pure deionised water. Over a period of 7 days of continuous operation, the composition of the rinse bath changes significantly. As a result, the rinse bath must be renewed weekly. Typically, tin and chloride ions, as well as hydrochloric acid (HCl), are carried over via the liquid film adhering to the substrates. However, even small quantities of palladium present in the liquid film on the substrate surface are carried into the rinse bath. As the rinse bath has a continuous inflow and outflow, this results in a significant loss of dissolved palladium.

Conventional palladium recovery processes from solutions typically make use of precipitation reactions [4,5,6], electrochemical methods [7,8,9], membrane separation techniques [10] and ion exchange resins [11,12,13]. Most methods are limited by, e.g., high operational costs, the need for complex equipment, the use of expensive and potentially hazardous reagents, high energy input or the inability to recover palladium from solutions with very low concentrations. In principle, the use of ion exchangers can enrich precious metals by a factor of 100 or even more. However, in the case of water containing high levels of non-ferrous metals, the ion-exchange resin becomes saturated quickly. As the waste water from the rinse bath in question contains significant quantities of tin, recovery using ion exchange resins is not economically viable. The resin would quickly become saturated with tin and would need to be replaced. Preliminary tests on conventional ion exchangers carried out by Hansgrohe did not yield any economically viable results [14]. In precipitation processes, metal cations are converted into their hydroxides by means of neutralisation precipitation. The sludge produced in this process can be filtered off or fed directly into a smelting process. However, the waste water in question must not consist of a mixture of multiple substances. If there are other metals in the solution alongside the precious metal, this can result in a mixture of different precipitation products, leading to more costly treatment of the sludge. The addition of flocculants does not contribute to the cost-effectiveness of the process either, as this can lead to a drastic increase in the volumes of wastewater and sludge. Cementation processes which result in the precipitation of palladium through the addition of base metals involve complex, time-consuming process steps that require the wastewater to be treated in batch operations [15]. However, if the wastewater is generated continuously and in large quantities, recovering the palladium using such a process was internally found to be not cost-effective [14].

In summary, it was therefore not possible to recover palladium economically at Hansgrohe, either by ion exchange or by precipitation, as the electroplating effluent from the sink in question has a low content of valuable metals and exhibits the characteristics of a multi-component mixture.

**Figure 1:**
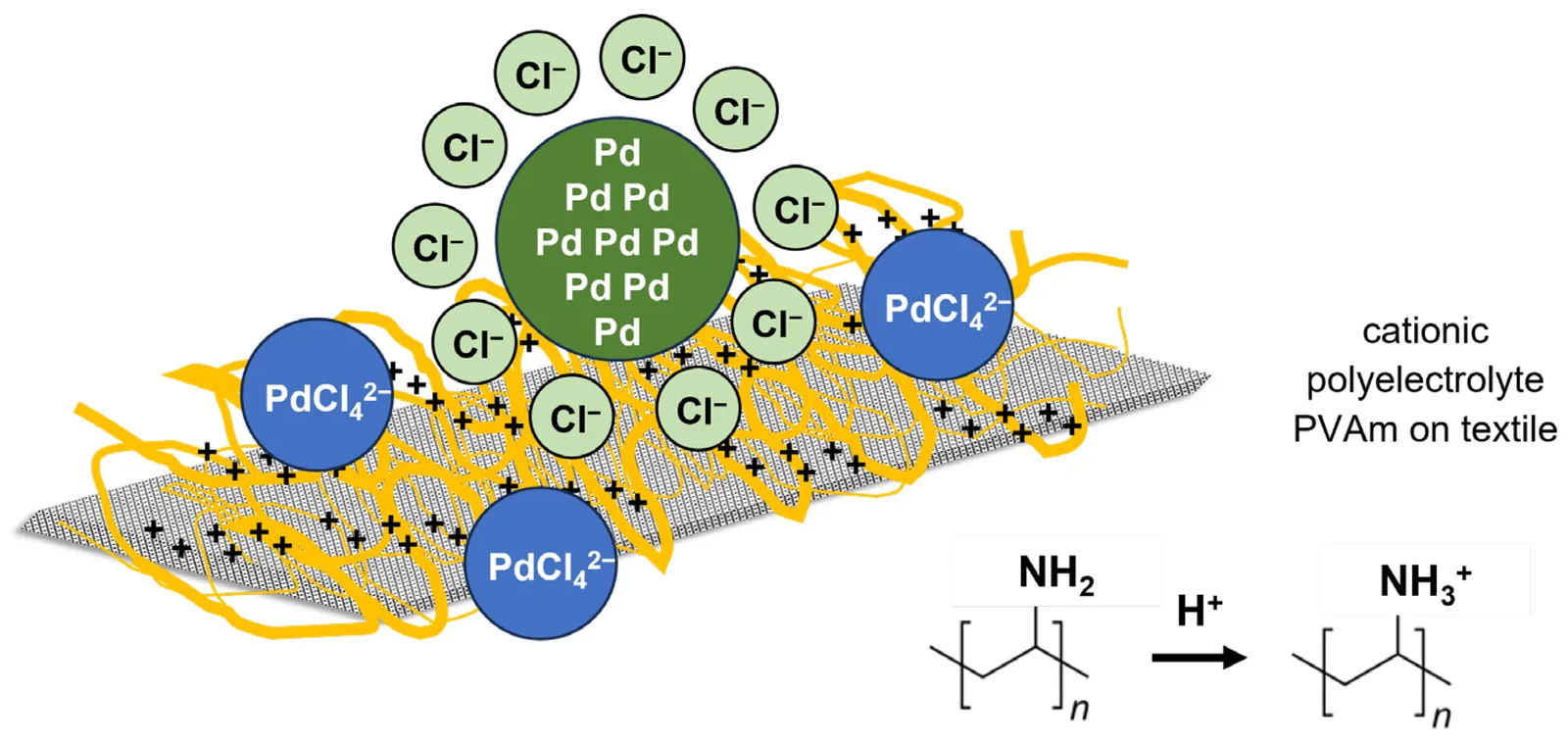
Schematic representation of a PET nonwoven fabric treated with PVAm and the binding of palladium ions [24].

In addition to the methods already mentioned, new adsorption materials and methods promise good recovery rates, at least on a laboratory scale. These include the use of activated carbon, mesoporous silicates, polyethers, nonionic polymers, or biopolymers, such as chitosan and cellulose [16,17,18,19,20,21]. Another novel adsorption material was developed at the DTNW and could offer an alternative to existing precious metal recovery methods. This process uses textiles on whose surface the cationic polyelectrolyte polyvinylamine (PVAm) is immobilized. The textile allows liquids to flow through it easily and is capable of enriching palladium from acidic aqueous solutions in the form of the anionic species PdCl_4_^2−^ as well as negatively charged colloidal palladium particles (Figure 1) [22,23,24].

In the printed circuit board industry, this system has already been successfully tested on wastewaters containing high concentrations of palladium in the range of 0.5–1.0 g/L [24]; however, it should be noted that there are significant differences in the physicochemical properties between the wastewater generated in that industry and the process water under consideration here.

The aim of this study was therefore to investigate whether PVAm-functionalized textiles can also be used for the recovery of palladium from low-concentration, tin-rich process water generated in the POP (Plating On Plastic) process under industrial operating conditions. This should be possible by expanding on our previous approach [22,24], first selectively separating the highly concentrated tin fraction so that, in the next step, we can enrich the low-concentration palladium fraction on the adsorbent fabric and subsequently recover it.

## 2. Results

### 2.1. Development and Production of a Textile Adsorber Material for Palladium

Based on the DTNW’s expertise regarding the immobilization of different polyelectrolytes, a two-step thermal fixation of the polyvinylamine combined with a subsequent crosslinking of the polyelectrolyte layer on the textile was explored. First, the process was optimized on lab-scale with regard to the key parameters of the polyvinylamine coating, the number of amino groups available for subsequent Palladium desorption and wash permanence.

**Figure 2:**
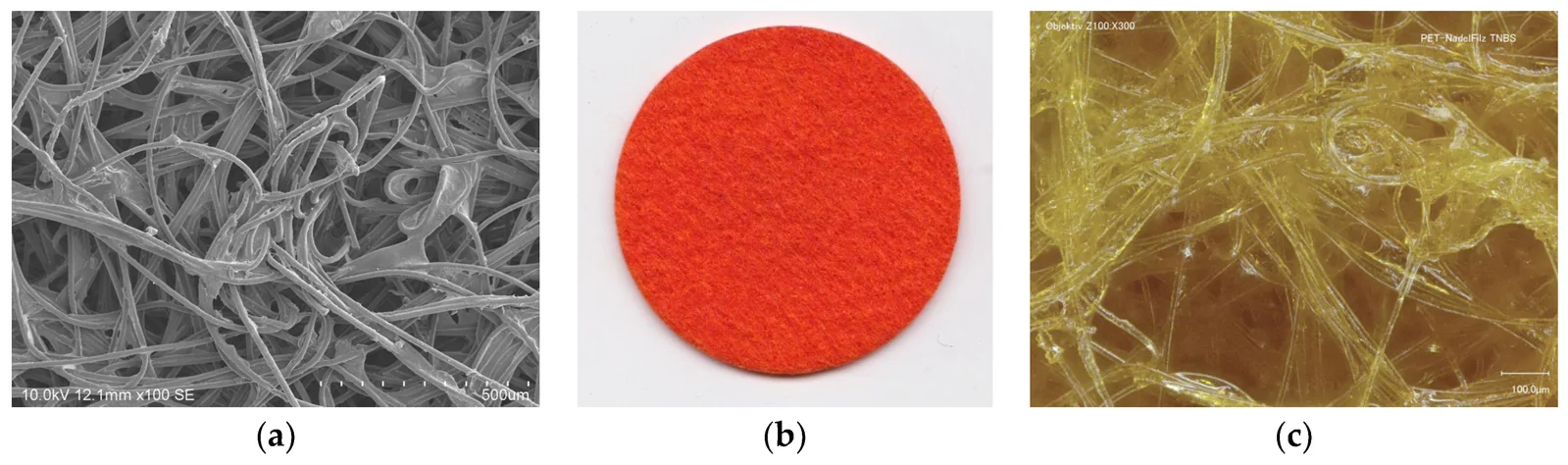
Scanning electron microscopy image (**a**) of a needle-punched felt made of polyethylene terephthalate (PET) after coating with polyvinylamine (PVAm). Photograph (**b**) and light-microscopic image (**c**) of the textile after staining with Orange II.

Figure 2a shows a scanning electron microscope image of the treated textile. The wash-resistant polyvinylamine coating on the fibres, resulting from partial cross-linking, is clearly visible. In addition, uncross-linked amino groups—which are therefore freely available for subsequent metal adsorption—can be visualised by staining with Orange II. The intensive staining after finishing can be clearly seen, demonstrating successful establishment of numerous amino groups on the textile surface. Figure 2b,c show a corresponding photograph as well as a light-microscope image of the stained adsorbent textile.

**Figure 3:**
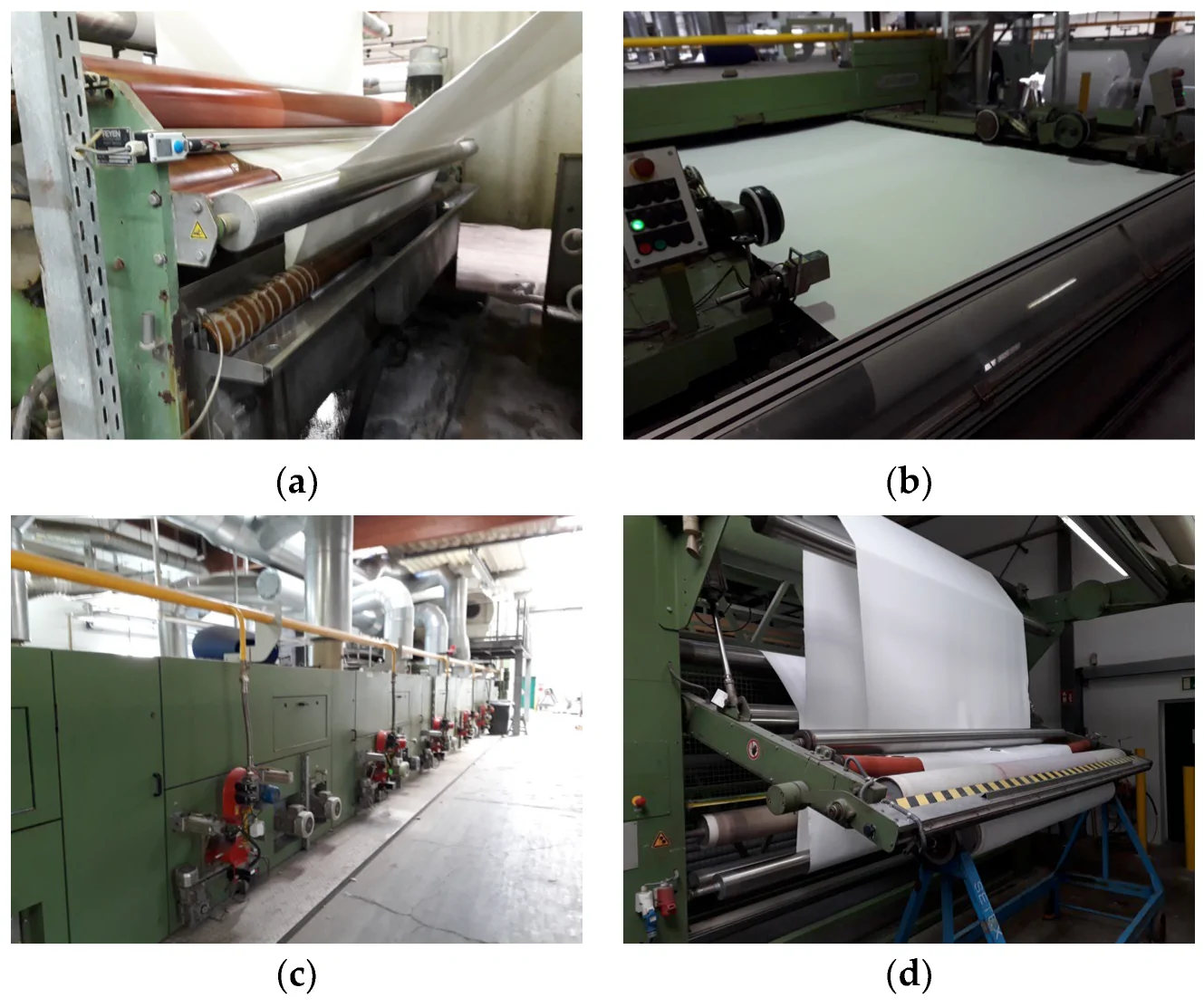
Industrial coating of polyester fibres with polyvinylamine. Fabric entry into the foulard and wetting (**a**), fabric entry into the tension frame (**b**), passage through the tension frame and thermal setting (**c**), fabric exit and winding (**d**).

After optimization, the fabric was finished with the commercially available polyvinylamine product Xelorex RS 1300 (BASF, Ludwigshafen, Germany, polymer content 20–22% by weight) in a two-step process on an industrial scale. In the first step, the fabric is passed through the PVAm solution, squeezed to a defined moisture absorption of 200%, and thermally fixed at 170 °C as it passes through a tensioning frame. This was followed by a second pass, during which the fabric and the PVAm layer were treated with the commercially available crosslinker Ruco-Coat FX 8011 (Rudolf, Geretsried, Germany) (liquor uptake 160%). The cross-linking reaction takes place at 170 °C. After thermal fixation, the fabric was cold-washed once to remove excess finishing chemicals. The average application rate of the finish is 25 percent by weight. The uniformity of the finishing was randomly assessed and confirmed at various points through Orange II staining. Figure 3 shows photographs of the industrial finishing.

**Figure 4:**
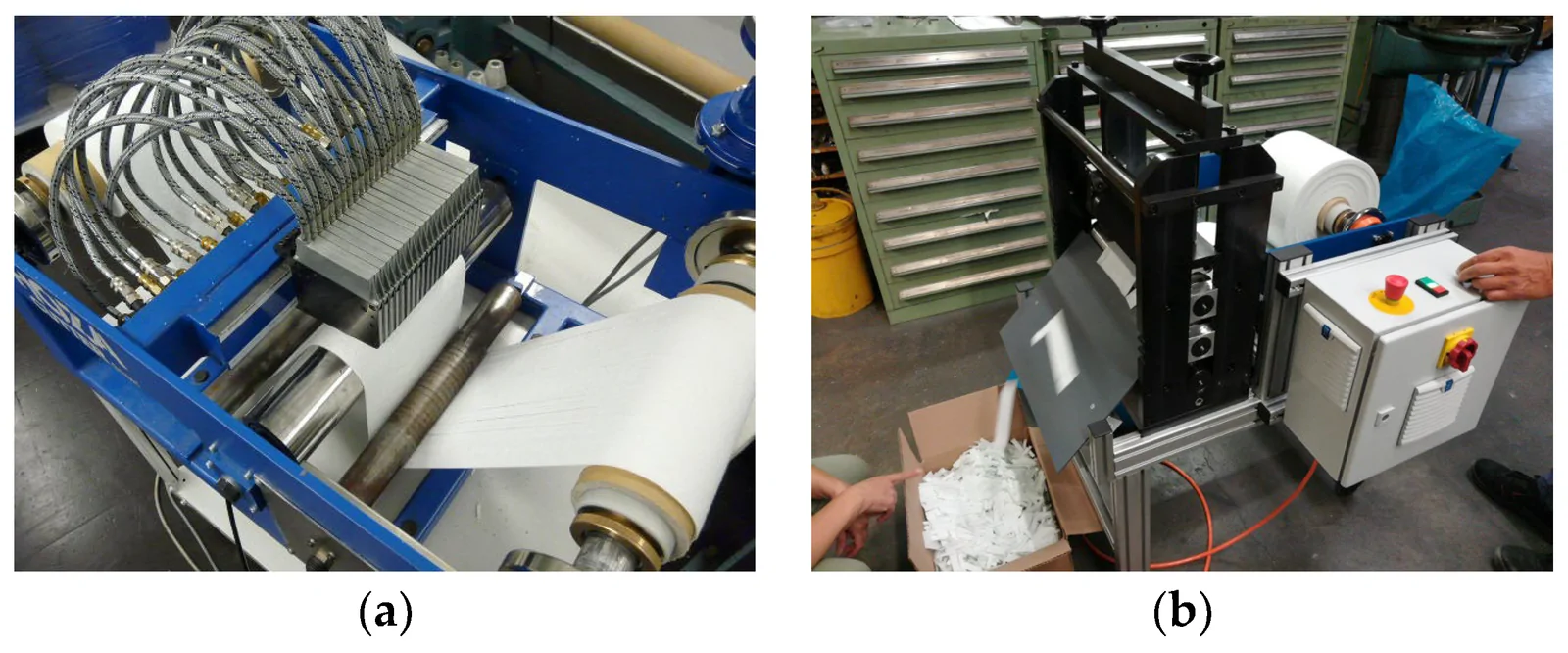
Preparation of the adsorbent fabric for further use. Cut to a width of 1 cm in a continuous strip (**a**), cut 5 cm × 1 cm (**b**).

The subsequent fabrication of the adsorbent textile was carried out on a semi-technical scale at the DTNW. The textile was first cut into rolls 20 cm wide, which were then cut into continuous strips 1 cm wide using a machine specially designed for this purpose (Figure 4a). Finally, the textile strips were cut into pieces measuring 5 cm × 1 cm (Figure 4b).

### 2.2. Pre-Study on the Selective Adsorption of Palladium from Industrial Process Waters onto Textiles Coated with PVAm

From our previous work [24], we know that up to 25 g of Pd can be adsorbed onto one kilogram of adsorbent fabric from highly concentrated Pd solutions. The intention of a first individual landmark experiment was to clarify the extent to which selective Pd enrichment is possible even at low concentrations and in the presence of high concentrations of interfering Sn ions, since the adsorption performance can be significantly impaired by the low initial concentration and competing ions [25].

At the DTNW, this exploratory experiment was carried out using PET needle-punched felts industrially treated with polyvinylamine to study the continuous adsorption of palladium from low concentrated rinse water (by Hansgrohe). The initial Pd concentration was 1.24 ± 0.12 mg/L (n = 6), the Sn concentration 734.2 ± 19.5 mg/L (n = 3) and the pH value 1.0.

**Figure 5:**
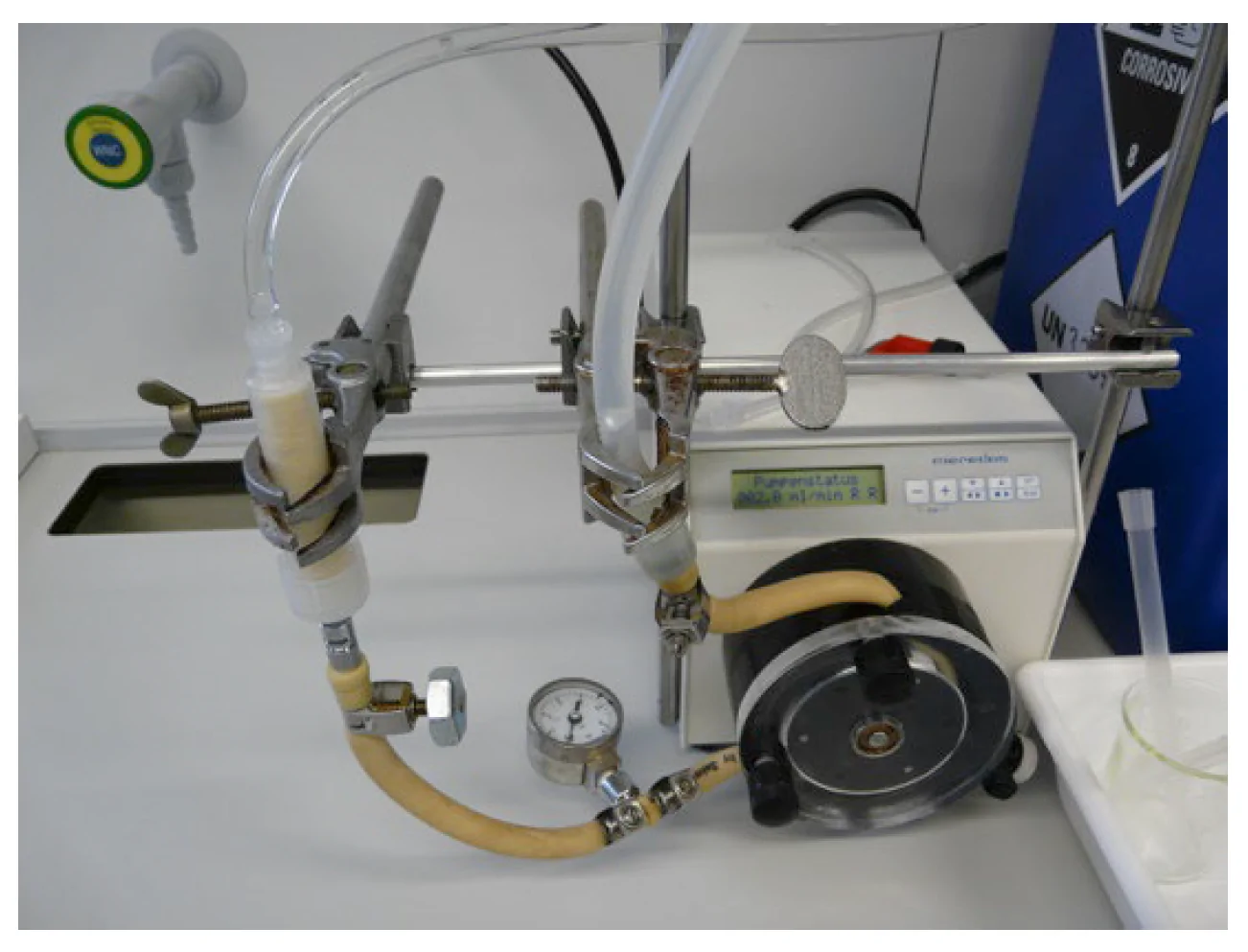
Apparatus for the continuous adsorption of palladium from industrial process water using adsorbent textiles on a laboratory scale.

We have used a densely packed 7 cm column containing 80 precisely defined round die-cut pieces (diameter 12 mm), which allows the adsorbed Pd loading on the textile to be determined at specific locations (experimental set-up shown in Figure 5, 2.2 g textile, flow rate: 0.85 L/h, Empty Bed Contact Time (EBCT): 33 s).

**Figure 6:**
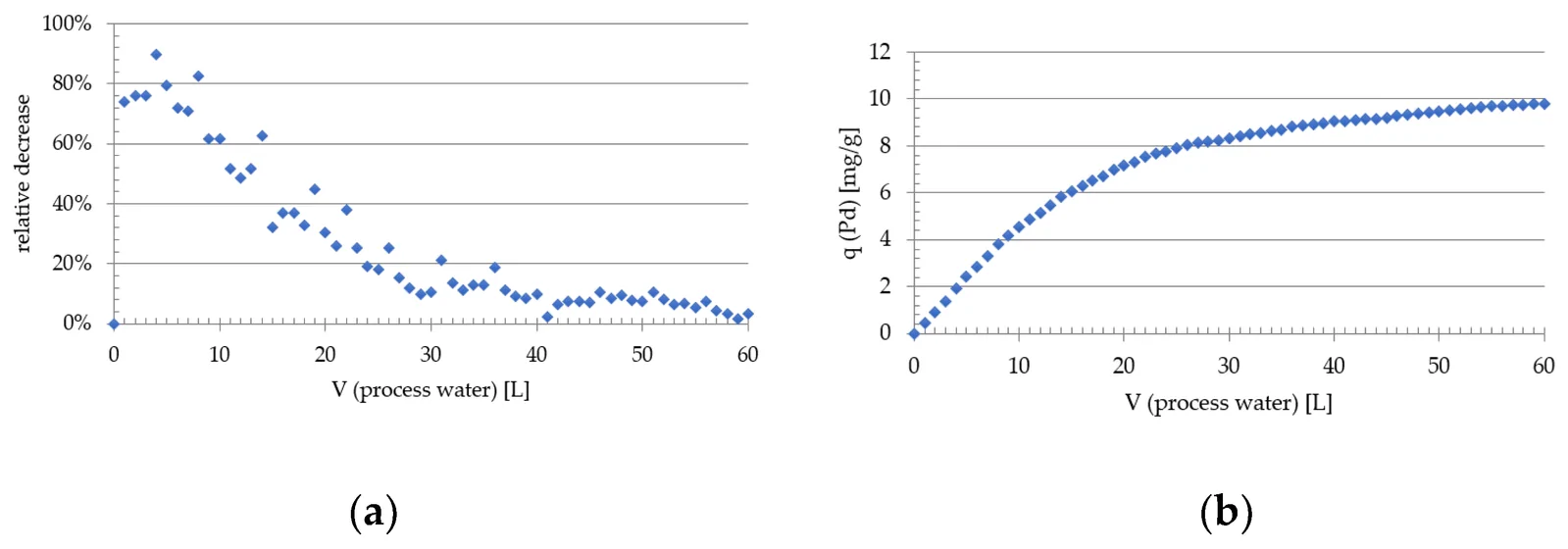
Relative decrease in the Pd content (**a**) of water containing Pd/Sn following adsorption on an adsorbent fabric on a laboratory scale using the adsorbent textile according to set-up shown in Figure 5 and calculated palladium enrichment on an adsorber fabric when water containing Pd/Sn flows through it (**b**).

By comparing the Pd concentration in the effluent after the solution had passed through the column with the initial Pd concentration, it was possible to calculate the relative depletion (Figure 6a) and the resulting enrichment on the textile (Figure 6b). This clearly demonstrates that the textile exhibits an adsorption capacity for palladium.

Once the experiment was complete, the column was opened and the textile discs were removed. Microwave-assisted digestion was carried out in suitable acids from various positions in order to dissolve the textile, together with the polyvinylamine coating and the bound metals. The palladium and tin contents of the textile were then quantified using ICP-OES (Table 1). Due to the significantly lower initial Pd concentration—and as expected—we did not achieve the high adsorbent capacity of our first study, which reached up to 25 mg Pd/g adsorbent textile [24]; however, with a loading of 7–8 mg Pd/g textile, it was clearly demonstrated here that Pd enrichment on the textile is possible even from very low-concentration aqueous solutions, even in the presence of a very high tin concentration. This potentially enables the cost-effective recovery of palladium from such wastewater and forms the basis for further R&D work at Hansgrohe, which is described below.

### 2.3. Technical Implementation for Palladium Recovery from Low-Concentration Rinse Water in the Direct Metallization Process for Plastics at Hansgrohe

Palladium-tin colloids are used as activators for the direct metallization of non-conductors, such as polyacrylonitrile-butadiene-styrene (ABS). These colloids generate a sufficiently electrically conductive surface, which is crucial for the subsequent metallization process. The palladium contained in the activator is carried into the rinse water through mechanisms such as entrainment, among others. Typically, the rinse water is then routed to wastewater treatment, and the valuable palladium is disposed of unused via the electroplating sludge.

Depending on the subsequent steps, the palladium content and the colloid size in the activators vary significantly. In the so-called direct metallization process, the tin bound in the colloids is substituted with copper and thus deposited electrolessly on the surface of the component. The subsequent electrolytic process originates from the cathodic contact on the component and therefore requires a closed network of palladium colloids. Consequently, a correspondingly high initial concentration in the activator is required. In the second variant, chemical nickel plating, the tin components first dissociate from the colloids, while the palladium remains on the surface. Subsequently, a thin nickel/phosphorus layer is deposited on the exposed palladium nuclei (chemical nickel plating). The growth of the nickel-phosphorus layer is autocatalytic. As a result, the layer grows homogeneously across the component surface from the individual catalytic centers. Consequently, this approach requires fewer nucleation sites and, therefore, lower palladium concentrations in the activators [2,3].

A high palladium concentration in the activator baths consequently leads to higher concentrations in the subsequent rinse cascades. To avoid compromising the rest of the plating process, the rinses are kept highly diluted and acidic through a high fresh water supply. These measures prevent the precipitation of tin(IV) oxide. Experience has shown that the concentration levels in the rinse stage are 1–3 ppm palladium and 700–900 ppm tin.

The textile adsorbent material described above was then used to selectively recover low-concentration palladium from strongly acidic, tin-containing rinse water. To this end, Hansgrohe developed a corresponding process technology, which is described below.

**Figure 7:**
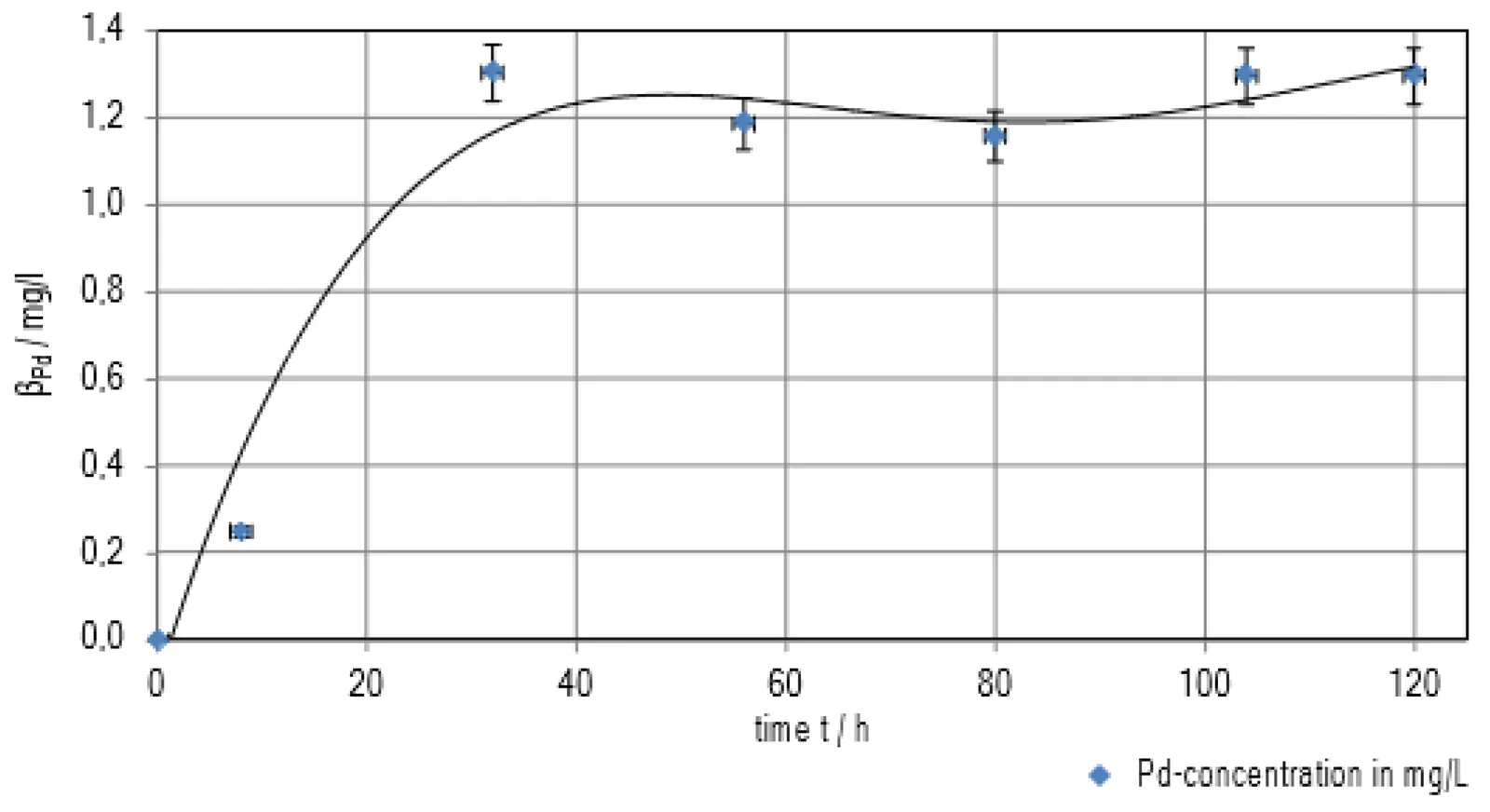
Changes in palladium concentration in the rinse tank.

The determination of palladium concentration in the first rinse tank downstream of the activator, conducted throughout the process, yielded the concentration curve shown in Figure 7. It should be noted that the rinse solution is replaced weekly, thereby regularly reducing the concentration. The concentration curve reaches saturation after approximately 48 h. At this point, equilibrium is achieved, with the input equal to the output.

An additional analysis of the rinse water using dynamic light scattering (DLS) revealed an average colloid size of approximately 490 nm. The particles contained in the rinse water were also examined using a high-resolution transmission electron microscope (HRTEM) with integrated EDX mapping. This analysis showed that the particles are composed of smaller agglomerated nanoscale formations. The EDX mapping shows that the particles consist mainly of tin (>97%) and only a very small proportion of palladium (<3%) [26].

#### 2.3.1. Step 1—Separation of Tin

Due to the high tin content of the particles, an upstream process step was defined to separate the tin-containing agglomerates from the finely particulate and ionic palladium. In addition to separating the tin, this prevents the mechanical blockage of the textile adsorbent material caused by precipitated tin. Without this pretreatment step, tin precipitation within the adsorption columns could significantly impair hydraulic performance and reduce the effective utilization of the adsorbent media.

Table 2 summarizes a one-time experiment designed to provide a rough estimate of the effects of pH increase and filtration on the prevailing concentrations. The concentrations of palladium and tin were compared both in the unfiltered state and in the permeate after filtration using a 0.45 µm filter. The effect of increasing the pH of the rinse water from 1.1 to 2.0 was also examined in both cases.

At an untreated pH value of 1.1, the concentrations of Pd (minus 13%) and Sn (minus 15%) change uniformly as a result of the separation of insoluble components through filtration. In contrast, raising the pH to 2.0 results in a significant reduction in the tin content in the permeate by 98%, while the palladium concentration is reduced only in the range of 30%. The pH adjustment promotes the formation of tin agglomerates, which are retained by the filter media and thus cause the tin concentration to decrease. Nevertheless, the precipitation of the tin component leads to undesirable co-precipitation of the palladium component. This must be taken into account during industrial implementation and, for example, minimized as much as possible through a gentle adjustment of the pH value. Further increasing the pH value above 2 is not recommended, as increased agglomeration of tin also results in more palladium being incorporated into the agglomerates and thus removed from the forced recovery process.

#### 2.3.2. Step 2—Separation of Palladium

**Figure 8:**
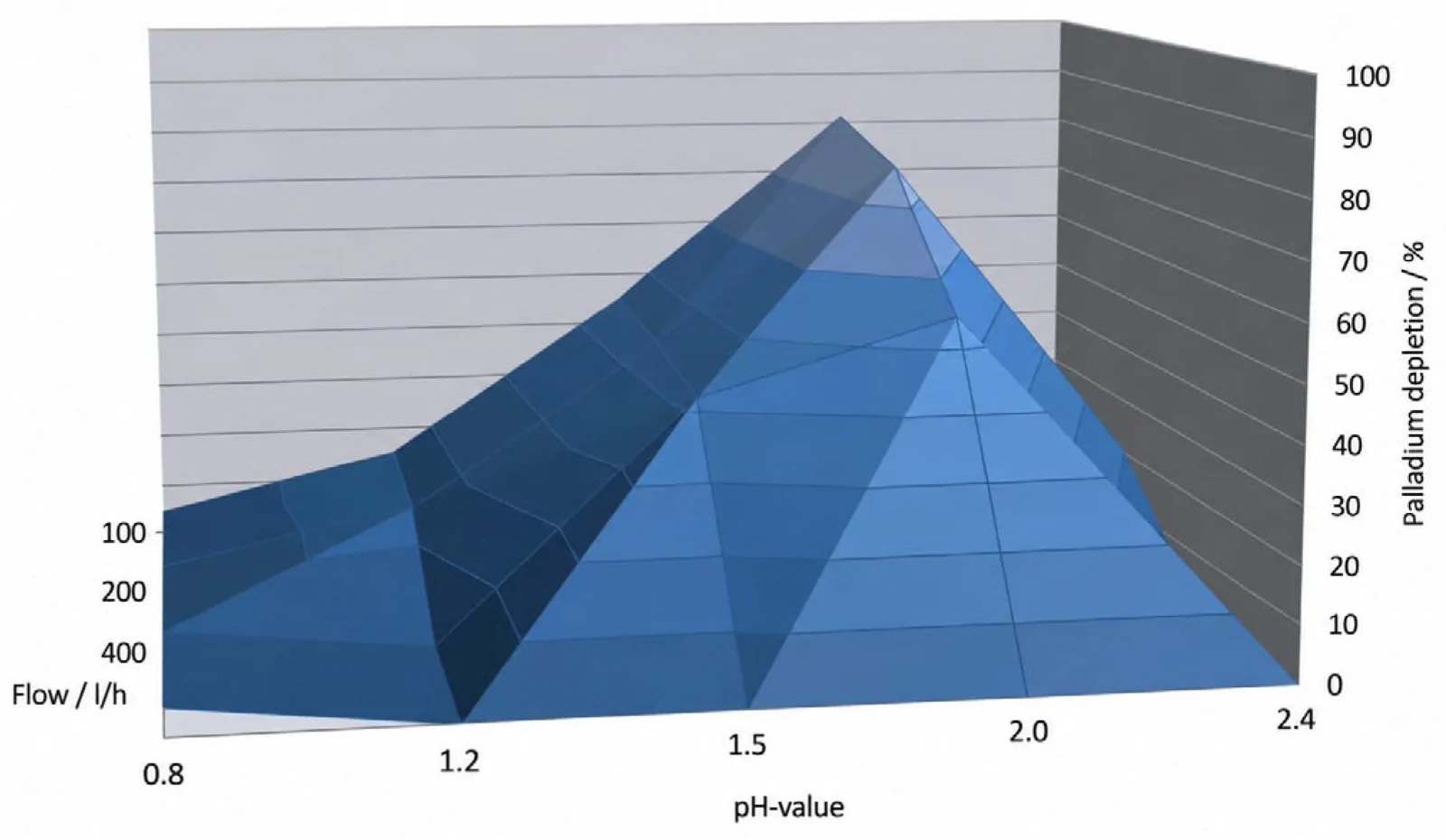
Dependence of palladium depletion on pH value and flow rate.

The next step in the process is the removal of palladium by the aid of the PVAm-modified adsorbent textile. The rinse water was passed through a defined volume of the textile material at various flow rates, and the corresponding depletion rate was determined at different pH values. Figure 8 shows the optimal operating conditions at a pH of 2 and a surface feed rate of 10 m/h.

In summary the following conclusions can be drawn:

▪ pH-controlled filtration can be used to remove tin
▪ At a pH of 2.0, the adsorbent fabric exhibits its maximum adsorption capacity for palladium

#### 2.3.3. System Design and Plant Operation

**Figure 9:**
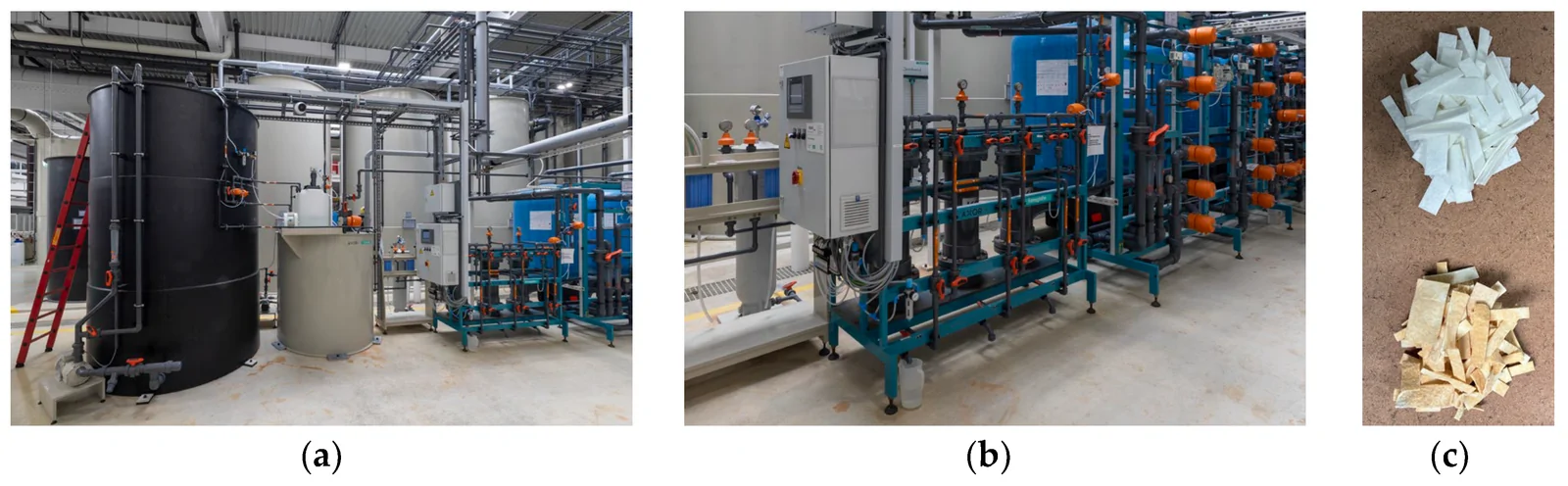
Photographs of the industrial plant for palladium recovery at Hansgrohe. (**a**): overall view with 8.5 m^3^ collection tank for rinse water containing Pd and 2.5 m^3^ Pd pretreatment stage with agitator and NaOH, (**b**): three columns 3 each with a volume of 25 L and filled with 1.5 kg of adsorbent textile, (**c**): blank adsorbent textile and adsorbent textile after Palladium adsorption.

Based on these key findings, a plant design was developed that enables the recovery of palladium from rinse water. Photographs of the industrial plant at Hansgrohe are shown in Figure 9. The entire process for the recovery of palladium from rinse waters consists of several individual steps. During the evaluation of all palladium-containing strands, the drip water was identified as a significant mass flow with high concentrations. In the electroplating process, the drip water is discharged separately via drip-off fixtures and, in the first process step, passed through a cartridge filter to retain the particulate matter it contains. The filtered drip water is then mixed with the contents of the process sink. Conditioning to pH 2 is achieved by adding caustic soda (NaOH). The tin agglomerates are separated by a pleated filter with a pore size of 0.5 µm to prevent unwanted competitive adsorption and filter blockage. The permeate is passed through the adsorbent textile, where the remaining palladium is then adsorbed. The process water, which is low in palladium, can now be fed into the standard wastewater treatment system for the electroplating process. The palladium-loaded textile was externally combusted and the pure Palladium can be recovered by a subsequent metallurgical processing.

The system treats a total rinse water volume of 5500 L per week, in addition to a continuous wastewater flow of 200 L/h. Based on an operating schedule of 15 shifts per week, each lasting 8 h, an additional 24 m^3^ of continuous wastewater is generated per week. The weekly operating cycle has been optimized to suit the conditions at the Hansgrohe site in Offenburg and is divided into two different plant operating modes.

First, the highly concentrated drip water is collected in a separate tank and mixed with the drained rinse water in the recovery system at the end of the week. Then, the system operates in batch mode (closed-loop circulation) from Saturday onward, following maintenance of the main production line and discharge of the 5500 L into the feed tank of the recovery plant. The batch operation continues until Tuesday. During this time, a large portion of the palladium is adsorbed onto the textile. Subsequently, the system is switched to continuous flow operation. At this point, the scrubber is already near saturation and has reached its maximum palladium concentration. As a general rule, the higher the palladium concentration, the higher the adsorption pressure on the exchange medium, and the more effective the separation of the palladium. Continuous operation is maintained until the scrubber is drained again at the end of the production week.

At an operating flow rate of 0.2 m^3^/h, the system provides an Empty Bed Contact Time (EBCT) of 6.6 min. Assuming a filter media porosity of 90%, the resulting hydraulic residence time is 5.94 min. This is an EBCT that is 12 times higher than that observed in our initial laboratory-scale experiment (see Section 2.2), so that a Pd loading higher than 7–8 mg Pd/g textile could be expected in advance. The system is operated 24 h per day. The palladium concentration is analyzed daily at both the system inlet and outlet. If the palladium concentration at the outlet exceeds 80% of the concentration measured at the inlet, the adsorbent material is considered saturated and the column media are replaced. Regardless of analytical results, the column fillings are replaced after a maximum operating period of two weeks, which yields in an annual consumption of 240–300 kg adsorbent textile.

The results over a 5-week period for the pH adjusted and filtered water are summarized in Table 3. In batch mode (closed-loop circulation), a recovery rate of 82% was achieved, whereas in continuous operation, the recovery rate was only 22%. Considering the total volume depleted during this period, the overall recovery rate is 48%. A maximum loading of 16 g Pd/kg textile was achieved. This corresponds to approximately 5 kg Palladium per year at the Hansgrohe facility in Offenburg, Germany, with a current world market price of approximately $200,000.

For a more comprehensive economic analysis, the estimated annual operating costs must also be taken into account. These currently amount to approximately $40,000, including the costs associated with textile replacement. Operating costs consist primarily of the annual cost of textiles (approx. $25,000), electricity costs (approx. $5000), and other supplies such as caustic soda (approx. $10,000). In addition, there are costs for the pyrometallurgical processing to produce pure palladium. These amount to approximately $20,000.

In addition, our system helps to protect the climate. According to a study by the International Platinum Group Metals Association on the life cycle assessment of platinum group metals, the greenhouse gas emission potential is approximately 28,000 kg CO_2_e per kg of palladium arrived from primary mining respectively 140 t/5 kg Pd [27]. For our recovery system, the electrical energy demand for the recovery of 5 kg Pd was estimated based on the installed power of the main pumps and agitators. Assuming continuous operation, this results in an annual electricity consumption of approximately 30,600 kWh/year and associated emissions of approximately 1.22 t CO_2_e/year, using an emission factor of 0.04 kg CO_2_e/kWh for German renewable electricity. This corresponds to a reduction in CO_2_ emissions of more than 99% compared to primary production.

## 3. Discussion

The processing and recovery of high-value precious metals such as platinum, gold, palladium, and silver—as well as strategic metals such as indium, gallium, niobium, tantalum, and the rare earth elements—from secondary raw material sources will become increasingly important in the coming years, particularly in countries with limited natural resources (“urban mining”). Accordingly, there is an increasing need to identify new, potentially usable sources of secondary raw materials while simultaneously developing cost-effective, energy-intensive processes to isolate and recover the relevant metals as selectively as possible from the given matrix.

The metallization of plastic objects produces particulate-laden rinse water containing very low concentrations of palladium and, at the same time, comparatively high concentrations of tin. Separating and recovering the palladium has so far proven difficult and not cost-effective. The palladium recovery concept developed here begins with a precise analysis of the target and interfering ion concentrations, as well as the particle shape and size. Subsequently, a multi-step process was developed for the selective separation of the fractions, consisting of rinse water collection, separation of the interfering ions (tin) via pH-based separation and filtration, as well as the adsorption and enrichment of the target metal (palladium) on a polyelectrolyte-functionalized adsorbent fabric. The results were scaled up and transferred to a pilot plant. This enabled us, for the first time, to demonstrate on an industrial scale that the recovery of palladium from low-concentration rinse water in the electroplating industry is both effective and cost-efficient. The purification of the palladium into its pure form is achieved through a pyrometallurgical process. In total, this enables the recovery of up to 5 kg of palladium annually at the Hansgrohe site in Offenburg, with a current material value of approximately $200,000—corresponding to a recovery rate of up to 48% for the pretreated water. If we take all operating expenses into account in this initial estimate, the net profit comes to approximately $140,000. At the same time, compared to primary production (mining) a reduction potential of approximately 140 metric tons of CO_2_ equivalents per year was achieved.

To date, pH adjustment and Sn filtration have been accompanied by undesirable co-precipitation of Pd of up to 30%, which reduces the overall recovery rate to 34%. However, as can be seen from the high recovery rate of 82% in close-loop mode, further optimizations can lead to even higher overall efficiencies. Currently, optimizations are underway to decrease the Pd loss during pretreatment, increase the contact time between the rinsing water and the textile, increase the number of Pd-binding amino groups on the textile surface, achieve even more precise pH control, and optimize the packing of the textile in the column.

Furthermore, we see exceptionally high potential for value creation at other Hansgrohe locations, at other companies with similar production processes, and throughout the electroplating industry as a whole. The adsorbent textile itself can, in principle, also be used across industries for the recovery of other precious metals or for the decontamination of hazardous metals such as chromium or arsenic from contaminated sites. Finally, we envision the use of our technology in the extraction of primary raw materials—for example, from mine drainage or surface waters.

## 4. Materials and Methods

A PET needle-punched felt from Kayser Filtertech GmbH (Dueren, Germany) was used as the base material (polyethylene terephthalate Type 111E90-000-0/0 mass per unit area 200 g/m^2^, strapless, heat-set). Textile finishing on laboratory, semi-technical and industrial scales was carried out by wetting the base textile with a homogenized mixture of 50% Xelorex RS 1300 (BASF, Ludwigshafen, Germany, polymer content 20–22% by weight) and 50% water with additional foulard squeezing (liquor uptake 200 w/w%). Afterwards a second passage was carried out with a homogenized mixture of 10% Ruco-Coat FX 8011 (Rudolf, Geretsried, Germany) and 90% water with additional foulard squeezing (liquor uptake 160 w/w%). The fixation temperature was again 170 °C, and the textiles were washed once after finishing.

Light microscopic images of the textile materials were taken with a VHX digital microscope from Keyence Deutschland GmbH (Neu-Isenburg, Germany). The scanning electron micrographs of the textile materials were obtained using a SEM S-3400 N II (Hitachi High-Technologies Europe GmbH, German representative: Krefeld).

The Palladium content of waters and solutions was quantitatively determined using an ICP-OES spectrometer (ICP/OES, Varian 720-ES, Kamp-Lintfort, Germany). For the quantitative determination of the Palladium loading of the textiles by ICP-OES, 200 mg of the textile was weighed in. The textiles were dissolved with 8 mL of 69% nitric acid in a microwave-activated reaction system (MARS5) from CEM GmbH (Kamp-Lintfort, Germany) at 800 watts (for up to 10 samples), or at 1600 watts (for 11 samples and more). The solution cooled down to room temperature was transferred with distilled water into a 25 mL volumetric flask and stored overnight in the refrigerator. After the sample solution had returned to room temperature, the flask was filled up to the mark with distilled water. Of the solution, 15 mL was filtered through a filter (0.2 µm). The respective metal content of the solutions thus obtained was quantitatively determined using ICP-OES.

## Figures and Tables

**Table 1:** Quantitative determination of the Pd and Sn content of the adsorber fabric following flow-through with water containing Pd and Sn.

Layer	Pd Content [mg/g Textile]	Sn Content [mg/g Textile]
1–6	8.27	0.22
19–24	7.42	0.06
40–45	6.81	0.12
58–63	7.59	0.04
76–81	7.68	0.11

**Table 2:** Pd concentration and Sn concentration of rinsing waters at various pH level.

Water	pH	Treatment	Pd [mg/L]	Sn [mg/L]
rinsing water	1.1	not filtered	0.68	254
rinsing water	1.1	filtered	0.59 (87%)	215 (85%)
rinsing water	2.0	not filtered	0.73	246
rinsing water	2.0	filtered	0.50 (69%)	5 (2%)

**Table 3:** Palladium recovery from rinsing waters using the industrial plant at Hansgrohe.

	Batch Mode			Continuous Mode		
	Input [mg/L]	Output [mg/L]	Depletion Rate	Input [mg/L]	Output [mg/L]	Depletion Rate
week 1	0.8	0.2	75%	1.6	1.4	13%
	0.6	0.05	92%	1.8	1.56	13%
week 2	1.3	0.2	85%	1.1	1.0	9%
	1.6	0.4	75%	1.31	0.98	25%
week 3	0.6	0.1	83%	0.67	0.36	46%
	1.0	0.2	80%	0.57	0.46	19%
week 4	1.9	0.3	84%	1.05	0.89	15%
	2.2	0.3	86%	0.6	0.43	28%
week 5	1.5	0.3	80%	2.2	1.5	31%
	1.3	0.3	77%	1.1	0.9	18%
mean value	1.28	0.235	82%	1.2	0.948	22%
standard deviation	0.51	0.1	0.05	0.51	0.42	0.11

## Data Availability

The raw data supporting the conclusions of this article will be made available by the authors on request.
